# A Dirichlet process model for classifying and forecasting epidemic curves

**DOI:** 10.1186/1471-2334-14-12

**Published:** 2014-01-09

**Authors:** Elaine O Nsoesie, Scotland C Leman, Madhav V Marathe

**Affiliations:** 1 Network Dynamics and Simulation Science Laboratory/Virginia Bioinformatics Institute/Virginia Tech, Blacksburg, Virginia, USA; 2 Department of Statistics/Virginia Tech, Blacksburg, Virginia, USA; 3 Department of Computer Science/ Virginia Tech, Blacksburg, Virginia, USA

**Keywords:** Dirichlet process model, Influenza epidemic, Simulations, Individual-based model, Epidemic forecasting

## Abstract

**Background:**

A forecast can be defined as an endeavor to quantitatively estimate a future event or probabilities assigned to a future occurrence. Forecasting stochastic processes such as epidemics is challenging since there are several biological, behavioral, and environmental factors that influence the number of cases observed at each point during an epidemic. However, accurate forecasts of epidemics would impact timely and effective implementation of public health interventions. In this study, we introduce a Dirichlet process (DP) model for classifying and forecasting influenza epidemic curves.

**Methods:**

The DP model is a nonparametric Bayesian approach that enables the matching of current influenza activity to simulated and historical patterns, identifies epidemic curves different from those observed in the past and enables prediction of the expected epidemic peak time. The method was validated using simulated influenza epidemics from an individual-based model and the accuracy was compared to that of the tree-based classification technique, Random Forest (RF), which has been shown to achieve high accuracy in the early prediction of epidemic curves using a classification approach. We also applied the method to forecasting influenza outbreaks in the United States from 1997–2013 using influenza-like illness (ILI) data from the Centers for Disease Control and Prevention (CDC).

**Results:**

We made the following observations. First, the DP model performed as well as RF in identifying several of the simulated epidemics. Second, the DP model correctly forecasted the peak time several days in advance for most of the simulated epidemics. Third, the accuracy of identifying epidemics different from those already observed improved with additional data, as expected. Fourth, both methods correctly classified epidemics with higher reproduction numbers (R) with a higher accuracy compared to epidemics with lower R values. Lastly, in the classification of seasonal influenza epidemics based on ILI data from the CDC, the methods’ performance was comparable.

**Conclusions:**

Although RF requires less computational time compared to the DP model, the algorithm is fully supervised implying that epidemic curves different from those previously observed will always be misclassified. In contrast, the DP model can be unsupervised, semi-supervised or fully supervised. Since both methods have their relative merits, an approach that uses both RF and the DP model could be beneficial.

## Background

Influenza pandemics result from influenza viruses capable of human-to-human transmission and for which a large global population has little or no pre-existing immunity [1]. A well known example is the 1918 pandemic, which resulted in an estimated 20–50 million deaths worldwide [2]. A similar pandemic today would likely result in higher morbidity and mortality due to increased travel within and between countries, increased urbanization and a growing aging and immunosuppressed population [3,4]. Reliable forecasts of public health measures (such as peak time, peak height and attack rate) could impact timely and effective implementation of interventions to limit the effect of a pandemic [5,6].

There were several endeavors towards real-time prediction of the expected peak time, peak height and attack rate during the 2009 pandemic. Examples include studies by Nishiura [5] and Ong et al. [7]. Nishiura [5] employed a discrete time stochastic likelihood-based model for forecasting epidemic dynamics in Japan, whereas, Ong et al. [7] proposed a Bayesian and stochastic compartmental model for real-time epidemic monitoring and forecasting in Singapore. In contrast to the likelihood and Bayesian methods, Nsoesie et al. [8] recently presented a supervised classification approach for predicting the epidemic curve (defined as the daily/weekly counts of infected persons or influenza-like illness (ILI) cases for the duration of an outbreak) based on the idea of matching ongoing influenza activity to historical and simulated influenza epidemic curves. The supervised classification approach presented by Nsoesie et al. [8] involved training the model on pre-defined groups of curves and all new curves were assigned to one of the pre-defined groups, which implies curves that are significantly different from those in the training set will always be misclassified.

**Figure 1:**
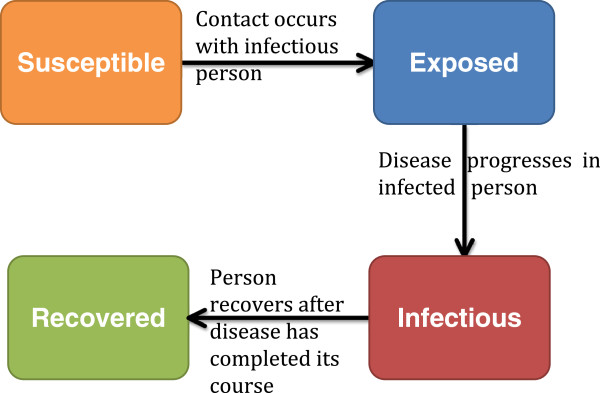
**SEIR model.** Description of the different compartments of an SEIR model.

On the other hand, we present a semi-supervised classification approach, which also involves training the model on pre-defined groups of curves, however, new curves are either classified into the pre-defined groups or a new group is created if the new curve is different. We explore the forecasting problem under the following scenario: during an influenza epidemic, several possible parameter sets are proposed for modeling the epidemic. Each parameter set consists of a transmissibility value (typically represented using the reproduction number), and the infectious and incubation periods. The hypothesized parameters are used to stochastically simulate possible scenarios that could describe the ongoing epidemic. We use a **S**usceptible, **E**xposed, **I**nfectious and **R**ecovered (SEIR) disease model as shown in Figure 1. We then create a library consisting of the simulated epidemics and historical epidemic data. All simulated epidemics with the same parameters are grouped. The parameters for a new epidemic are inferred by comparing the partial epidemic curve to the epidemic curves in the library. In the event that the new epidemic cannot be assigned to any of the groups in the library, a combination of expert opinion and search algorithms are used to propose parameters for modeling the epidemic. The simulation and classification process is repeated for each day/week *j* as data is updated. This study completes the first part of a two-step procedure for forecasting the epidemic curve using this approach. The first step of the procedure involves identifying epidemic curves similar or different from those in the epidemic library. The second step involves searching for parameters to model an ongoing epidemic if it is different from those in the library.

The individual-based model used in simulating the data in this study is a computational epidemiology model consisting of a disease model and a dynamical network with detailed representation of synthetic populations [9-11]. Disease is transmitted through contacts between susceptible and infectious individuals. Individuals move through four disease states (Figure 1): susceptible, exposed, infectious and recovered. Recovered individuals remain in the population but can no longer spread the disease due to immunity. To classify epidemics stochastically simulated using the individual-based model, we explore the grouping properties of a semi-supervised Dirichlet process model. The Dirichlet process is a nonparametric Bayesian procedure that presents a good solution to this problem since curves different from those in the library can be identified. Since Ferguson [12] formalized the Dirichlet process as a prior over distributions, there have been several extensions in terms of inference and applications [13]. The Dirichlet process has been proposed as a solution to finding the number of spatial activation patterns in fMRI images [14], the modeling of unknown number of topics across several corpora of documents [15], grouping population genetics data [16], detecting positive selection in protein-coding DNA sequences [17] etc. Although the Dirichlet process has been used in several studies, to our knowledge it has not been used in a procedure aimed at classifying and forecasting epidemic curves.

The premise of this paper is to validate the functionality of the Dirichlet process model for classifying and forecasting epidemic curves using model-generated epidemic data. The main aims of the study are therefore to: (i) compare the accuracy of the Dirichlet process model to that of random forest, which has been shown to achieve high accuracy in correctly classifying simulated epidemics based on the partial epidemic curve; (ii) forecast the epidemic peak time before the peak is observed and (iii) identify epidemic curves different from those in the library. Additionally, we also forecast influenza outbreaks in the United States from 1997–2013 based on ILI data from the Centers for Disease Control and Prevention (CDC). There are several advantages to using this simulation and classification approach for classifying and forecasting epidemic curves. First, the method captures the temporal trend of the epidemic curve. Second, the epidemic curve can be estimated under different scenarios by implementing changes to the individual-based model. Third, the approach identifies curves similar or different from those in the library.

### Epidemic simulation

The individual-based model used in generating the data in this study has been used for studying influenza dynamics and transmission, and evaluating control strategies [8,9,18]. The construction of the individual-based model involves the creation of a state-of-the-art behavioral model and a disease model. To create the behavioral model, a synthetic population for a specific geographic region is constructed using United States census data [19]. Each individual is assigned a set of demographic variables such as age, household size, household income etc. Households in the synthetic population are located such that a census of the synthetic population is statistically identical to the real census data at the block level [20]. Each synthetic individual is also assigned a schedule based on data from an activity survey [21]. Synthetic individuals come in contact with other individuals at different activity locations resulting in a dynamic social contact network through which disease transmission occurs. The individual-based model is not the focus of this study since similar data can be simulated using a compartmental SEIR model. However, we use the individual-based model since the overall aim of the project is to forecast the epidemic curve, and investigate the possible effects of interventions and changes in individual behavior during an outbreak. Details of the individual-based model are summarized in the Additional file 1. A detailed description can also be found in [9].

**Figure 2:**
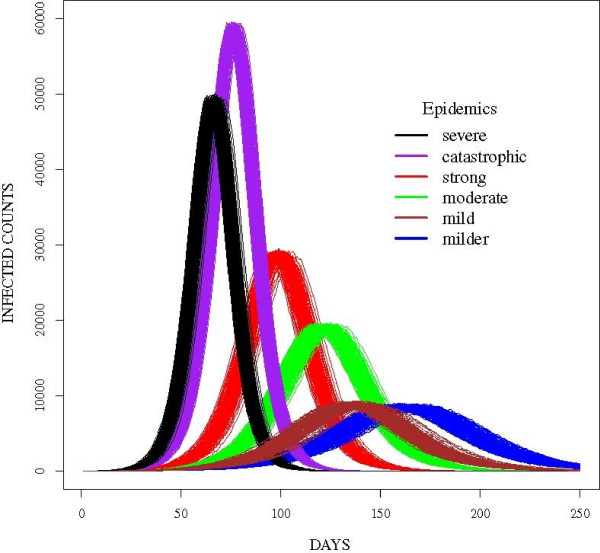
Sample epidemic curves for simulated epidemics in the study.

The data in this study was simulated using six parameter sets (Figure 2). The incubation and infectious period distributions were based on parameters used to model seasonal influenza epidemics [22] and the 2009 H1N1(A) pandemic [8]. Transmissibility values were selected to produce epidemics similar to and more severe than seasonal influenza. Each simulated epidemic was replicated two-hundred times to capture the underlying stochastic process. For the purposes of this paper, the epidemics were named *catastrophic, severe, strong, moderate, mild* and *milder*. Per Figure 2, the epidemics peaked at distinct times except for the mild and milder epidemics. The complexity of this study therefore involved differentiating these epidemic curves during the early stages of the epidemics. Hence, we aimed to develop a procedure which classifies epidemic curves and forecasts the peak in the early stages of the outbreak since earlier forecasts would be most useful to public health officials.

### Selection of a nonlinear model

During most infectious disease outbreaks that confer immunity upon recovery, the number of new cases increases until it peaks and then declines thereafter [23]. Under the assumption of a single peak, we modeled the simulated daily infected-counts using parametric models based on select statistical distributions. We used nonlinear models based on negative binomial, Poisson, Weibull, normal, Pareto, generalized extreme values (GEV) and Cauchy basis functions. The models were selected to allow exploration of different discrete and continuous distributions that appeared to capture different aspects of the epidemic curves. For example, the normal model was selected since it appeared to represent the shape of the simulated epidemic curves. On the other hand, heavy tailed distributions such as the Weibull, Pareto and Cauchy were selected to capture the heavy tails observed in some epidemic curves. In addition, since the epidemic curves were daily counts of infected persons over time, we also used models based on discrete distributions such as Poisson and negative binomial. Finally, the GEV, which is a three parameter (location, scale and shape) family distribution, was chosen to model deviations in the shape of the epidemic curves. We provide explicit details on the model formulation in a later section.

To illustrate the fits of the data to the selected models, we did the following. First, for each selected model, we estimated the parameters for each epidemic curve using the maximum likelihood procedure. Typically, the duration of an ongoing epidemic is unknown and this assumption was held for the remainder of the analysis. However, for illustrating the model fitting, we used a duration of approximately 180 days. Next, the estimated parameters were used to sample from the parametric model with the same length (number of days) as the epidemic curves. The epidemic curve was then normalized and compared to normalized samples from the parametric model. Lastly, the absolute error was estimated for each day and the mean absolute error was estimated across all days. The analysis was performed for each of the epidemic curves in the epidemic library. The average of the mean and variance of the mean absolute error were taken across all epidemic curves. The results are shown in Table 1.

**Table 1:** Fit of data to parametric models

**Models**	**Mean of mean**	**Variance of mean**
	**absolute error**	**absolute error**
Negative binomial	0.00070	2.56e-09
Poisson	0.00167	5.18e-07
Weibull	0.00118	2.25e-08
Normal	0.00058	3.18e-09
Pareto	0.00747	1.02e-06
GEV	0.00122	4.63e-08
Cauchy	0.00163	5.81e-09

**Figure 3:**
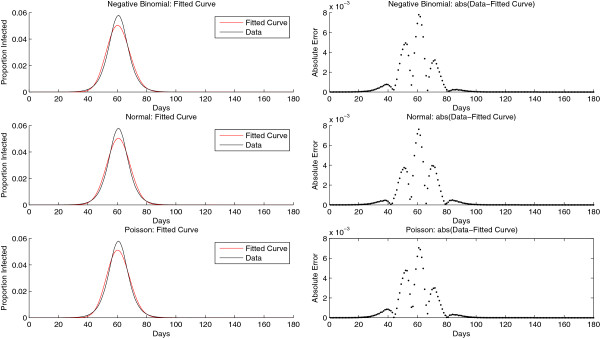
**A sample fit of a randomly selected epidemic curve to three parametric models.** The x-axis represents the days and the y-axis is the normalized number of infected persons relative to the cumulative infected. The black curve is the true epidemic curve and the red is the fitted curve. Using the epidemic data, the maximum likelihood procedure is used in estimating the parameters for each of the models. The normalized epidemic data is plotted against samples from the distributions based on the estimated parameters.

The normal, negative binomial and Weibull shapes had the best fit to all epidemic curves based on the mean of the mean absolute error of the fitted data to the observed data as shown in Table 1. The normal, negative binomial and Weibull were ranked best based on the variance of the mean absolute error. The shape of the GEV resulted in fits similar to that of the Weibull with estimated *k*<−0.5 implying the GEV distribution converged to the reversed Weibull distribution. In some cases, the best fit for each epidemic group was different from observations presented in Table 1. However, in most cases, models based on the normal and negative binomial distributions were ranked best based on the measures in Table 1. For the third best distribution, the selection lay between Weibull, the GEV and Poisson for most epidemic curves. The Poisson distribution was selected as the third parametric model for this analysis, because the beta distribution could be used as a conjugate prior to the Poisson density, enabling a closed form estimation of the posterior and predictive distributions. Sample fits of the normal, negative binomial and Poisson models to a randomly chosen epidemic curve are illustrated in Figure 3. Although the model fits in Figure 3 appear similar, in most cases, the fit of the parametric models varied depending on the shape of the epidemic curve.

## Methods

As stated, epidemic curves simulated using the same disease model parameters were assigned to the same group. In addition, each group also had a different set of parameters for the same family of distributions such as the normal distribution. Parameter references for the remainder of this section refer to the later. The number of possible groups and the parameters describing each curve were considered to be random variables. Furthermore, the prior probability distribution for the number of groups was described by a Dirichlet process prior. The Dirichlet process model was used in classifying epidemic curves and parameters in the fitted model were used in forecasting the epidemic peak.

### Dirichlet process models

The Dirichlet process (DP) represents a process prior in nonparametric Bayesian mixture models. Here nonparametric implies that distributions from the Dirichlet process have an infinite number of parameters [24]. The Dirichlet process can be defined as follows:

Let H be a distribution over a measurable space *Γ* and *α* a positive real number. For any finite measurable partition *B*_1_,…,*B*_*r*_ of *Γ*, the random vector (*G*(*B*_1_),…,*G*(*B*_*r*_)) has a finite-dimensional Dirichlet distribution with base measure H and concentration parameter *α* given by *G*∼*DP*(*α*,*H*) if:

(1) $$\begin{array}{l} \left(G(B_{1},\ldots ,G(B_{r}))∼\text{Dir}(\mathrm{\alpha H}(B_{1}),\ldots ,\mathrm{\alpha H}(B_{r})\right) \end{array}$$

The two parameters H and *α* denote the mean: *E*[ *G*(*B*)]=*H*(*B*) and inverse variance: *V*[ *G*(*B*)]=*H*(*B*)(1−*H*(*B*))/(*α*+1) of the DP respectively for any measurable partition B⊂*Γ*. Since *α* represents the inverse variance, when *α* is large, which implies a small variance, the DP is concentrated around its mean. As *α*→*∞* for any measurable B, *G*(*B*)→*H*(*B*). Additionally, draws from a DP are discrete since G is discrete with countably infinite point masses, even when H is small [25].

A simple property of a finite-dimensional Dirichlet distribution is that the sum of the probabilities of disjoint partitions is also a joint Dirichlet distribution whose parameters are sums of the parameters of the original Dirichlet distribution [13]. This property also holds true for the Dirichlet process. Moreover, samples from a DP are discrete, which leads to the observation of ties useful for grouping. The grouping property of the Dirichlet process can be best described using the Chinese restaurant process.

#### Chinese restaurant process

The Chinese Restaurant Process (CRP) can be described as follows: consider a restaurant with infinitely many tables and an infinite number of customers can be seated at each table. Each customer enters the restaurant and selects a table. In general, the (*n*+1)^*s* *t*^ customer would sit at an occupied table with probability proportional to the number *n*_*k*_ of customers at that table or sit at a new table with probability proportional to *α*. This can be represented as:

(2) $$\;\begin{array}{l} X_{n}|X_{1},\ldots ,X_{n-1}∼\\ \;\left\{\begin{array}{rrrr} {X_{k}}^{*} & \text{with probability} & \frac{n_{k}}{\alpha +n-1} & k=1,\ldots \\ \text{new draw from G} & \text{with probability} & \frac{\alpha }{\alpha +n-1} \end{array}\right) \end{array}$$

An important aspect of CRP, is the fact that most Chinese restaurants have round tables. This implies that with *n* customers in the restaurant, the tables would define both a distribution over permutations of *n* and a distribution over partitions *n*. The expected number of tables m among the n customers is given by:

(3) $$\begin{array}{l} E[\;m|n]=\overset{n}{\underset{k=1}{\sum }}\frac{\alpha }{\alpha +k-1}\in O(\alpha \text{log n}) \end{array}$$

Where *α*/(*α*+*k*+1) is the probability that the *k*^*t* *h*^ customer takes a new table. Note that the number of tables grows logarithmically in the number of observations. A large *α* will result in a large number of tables a priori.

### Semi-supervised Dirichlet process model

The classification problem was formulated using the CRP representation of the Dirichlet process. We used the epidemic curves and reference type for each epidemic group to represent customers and tables in the restaurant respectively. Note that all members of an epidemic group were assigned to the same table. At each point of classification, there were at most (k+1) tables, since a new epidemic curve was either classified to a pre-existing epidemic group or to a new one based on the posterior probability of belonging to each of the groups. Initially, we developed a Dirichlet process model for each of the three previously selected parametric distributions (normal, Poisson and negative binomial) and used a Markov Chain Monte Carlo (MCMC) procedure [26] for parameter estimation. After comparing the performance of the three models, we decided to use the normal model because the model fit well to the epidemic curve data and the model parameters were easy to interpret.

The normal Dirichlet process procedure was implemented in four steps. First, we grouped epidemics with the same disease model parameters (transmissibility value, incubation and infectious period distributions). Second, for each day *j*, for each group, we inferred normal model parameters using the slice sampling procedure. Slice sampling is also an MCMC method, which enables random sampling from probability distributions [27]. See Neal [27] for additional information. This procedure is equivalent to parameter estimation in a nonlinear regression framework.

The nonlinear regression model relating the daily infected counts for epidemic curve *j*, at time *t* (*y*_*j*,*t*_) was given by:

(4) $$\begin{array}{l} y_{j,t}=f(\theta ,t)+\varepsilon_{t} \end{array}$$

***θ*** was the vector of parameters and *ε*_*t*_ was the random error. *f*(***θ***,*x*) represented nonlinear basis function, which was a normal curve with parameters ***θ*** =*ϕ*,*μ*,*σ* given by:

(5) $$\begin{array}{l} f(\theta ,t)=\phi e^{\frac{-{\left(t-\mu \right)}^{2}}{2\sigma^{2}}}, \end{array}$$

where *ϕ* scaled the height of the function, *μ* was the mean of the function (representing the peak day), and *σ*^2^ modeled the variability of the epidemic curve. We defined *ε*_*t*_∼*N*(0,1/*γ*) and used standard reference priors for $p(\gamma )\propto \frac{1}{\gamma }$ and *p*(***θ***)∝1 to arrive at the posterior:

() $$\begin{array}{llll} p(\theta ,\gamma ) & \propto & \left(\overset{N}{\underset{j=1}{\prod }}\overset{T}{\underset{t=1}{\prod }}\gamma^{1/2}e^{-\gamma \frac{{\left(y_{j,t}-f(\theta ,t)\right)}^{2}}{2}}\right)p(\gamma )p(\theta ) & \\ & = & \gamma^{(N*t-2)/2}e^{-(\gamma /2)\overset{N}{\underset{j=1}{\sum }}\overset{T}{\underset{t=1}{\sum }}{\left(\;y_{j,t}-f(\theta ,t)\right)}^{2}}, & \end{array}$$

where *N* was the number of epidemic curves in each group and *T* was the total number of time points. Third, at each day *j*, we calculated the posterior predictive probability of a new curve belonging to each of the groups in the library. Last, we classified the new curve to one of the groups in the library or created a new group. We performed the prediction procedure independently for six hundred simulated epidemic curves.

Additionally, we forecasted the timing of the peak and evaluated the accuracy of the DP model in identifying epidemic curves different from those in the library. As stated, if an epidemic curve was different from the curves in the library, the DP model was expected to create a new group. The number of new groups created by the DP model is dependent on the choice of *α*. To select an appropriate value for *α*, we performed a sensitivity analysis by perturbing the value of *α* and measuring the prediction accuracy. We set *α* at 0.001 after comparing the error rates of higher and lower values.

### Forecast of peak time of epidemics in the U.S

We used both random forest and the DP model to forecast peaks of influenza outbreaks observed in the US from 1997–2013. Data on estimated percent ILI were obtained from the CDC influenza surveillance website (http://www.cdc.gov/flu/weekly/pastreports.htm). We divided the data into training and test sets. The training set consisted of yearly ILI data from 1997–2007 and the test set contained data from 2007–2013. Data for the first five influenza seasons started on week 40 in one year and ended on week 20 in the next year. For consistency, we defined the epidemic curve for each influenza season starting from week 40. The data in the training set was used to the train the random forest algorithm and the DP model, whereas data in the test set was used for predictions. Each of the ILI epidemic curves in the training set was placed in a separate group in the library and each epidemic curve in the test set was independently classified. We made predictions using sixteen, twenty-five and thirty-three weeks of data. The numbers of weeks were selected to make predictions before and after the peaks of most of the outbreaks, and towards the end of the influenza season. We evaluated accuracy of forecast based on prediction of the peak time.

## Results

The results are organized into three sections. In the first section, we discuss the results from classifying epidemic curves similar to those in the epidemic library. In the next section, we present additional features of the DP model; namely the forecasting of the epidemic peak time, and the identification of novel epidemic curves. Finally, we discuss the forecasting of the peak time of seasonal epidemics in the United States using percent ILI data.

### Comparison of DP model to random forest

**Figure 4:**
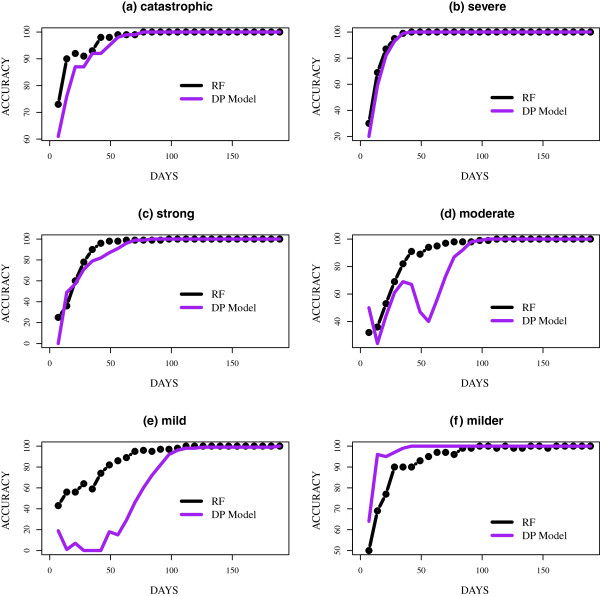
**Accuracy of predicting the epidemic curves in the test set.** The accuracy of predicting the epidemic curves in the test set: **(a)** catastrophic, **(b)** severe, **(c)** strong, **(d)** moderate, **(e)** mild and **(f)** milder. Accuracy on day *j* is the number of curves correctly identified based on the partial epidemic curve on day *j*. The results are presented based on epidemic peak time. The DP (Dirichlet Process) model is compared to RF (random forest).

The classification was performed at the end of each week starting from the first week to week twenty-seven since all the simulated epidemics peaked by the end of week twenty-seven (day 189). The accuracy of classifying the epidemic curves, defined as the percent of correctly identified epidemic curves on day *j*, given data on the number of daily infected up to day *j*, is presented in Figure 4. We presented the accuracy from day 7 to 189. For four out of the six groups, the accuracy of the methods was almost identical. Catastrophic and severe epidemics (Figure 4(a) and (b)) peaked sooner than the other groups and also had significantly higher peaks making them more distinguishable. In the classification of catastrophic epidemics, both methods reached over 90% accuracy on day 28, which was several weeks before the mean peak day of the epidemics. Similar to the identification of catastrophic epidemics, over 90% of severe epidemics were correctly identified several days before the mean peak day of the epidemics, which was observed on day 67. Strong and milder epidemics (Figure 4(c) and (d)) were also easily identified. The methods both surpassed 95% accuracy by day 63, which was approximately two weeks before the mean peak day of 98 for strong epidemics. For milder epidemics, the DP model commenced with a significantly higher accuracy, over 75% on day 7 and consistently improved over time. RF appeared unstable, which was likely due to the similarity between milder and mild epidemic curves.

Although the DP model performed extremely well in identifying previously discussed epidemic curves, the model encountered difficulties in classifying moderate and mild epidemics (Figure 4(d) and (e)). This was likely due to the fact that mild and moderate epidemics were sandwiched between strong and milder epidemics. Both methods appeared unstable in identifying these epidemics, although the instability was most evident in the DP model. In several instances, moderate epidemics were misclassified as strong and mild, while mild epidemics were misclassified as milder. However, as the epidemics neared their peaks, both methods could distinguish the curves from the other groups. Both methods had an accuracy of over 90% before day 84, which was several weeks from the mean peak day; day 121 for moderate epidemics. On the contrary, the DP model achieved over 90% accuracy around day 98 in the classification of mild epidemics, which had a mean peak day of 136.

The accuracy of classifying partial epidemic curves was higher for simulated epidemics with a higher reproduction number (*R*). The mean *R* at the start of the simulated epidemics were approximately 1.42, 1.45, 1.51, 1.59, 1.60 and 1.79 for milder, mild, moderate, strong, severe and catastrophic epidemics respectively. Both methods achieved significantly higher accuracy in the classification of catastrophic, severe and strong epidemics compared to moderate, mild and milder epidemics. In addition, the accuracy of both methods were more reliable and consistent as epidemics neared their peaks. This agrees with other published studies, which suggest that the accuracy of forecasting methods can be sensitive to the point at which forecasts are made [5,7,28].

### Additional features of the DP model

As stated, the DP model has additional features, which are not inherent in the RF approach. The DP model can be used to identify epidemic curves different from those in the library and forecast the expected peak based on parameters estimated using the slice sampling approach. On the other hand, RF is a fully supervised classification algorithm, which implies that epidemic curves that are significantly different from those in the library will always be misclassified. In addition, RF can only forecast the peaks of epidemic curves similar to those in the library.

#### Forecast of the peak time

Simulated epidemics in the various groups peaked within the following time ranges (days): severe [63–72], catastrophic [73–81], strong [94–106], moderate [116–128], mild [127–150] and milder [149–179]. Peak days falling within the true range for severe epidemics were forecasted on day 70 with a 95% credible interval of [67.385–67.726]. For catastrophic epidemics, the peaks were correctly forecasted on day 77 with a 95% credible interval of [78.964–79.544]. Peak timing for strong epidemics were correctly predicted on day 91 with a 95% credible interval of [104.42–105.08]. Moderate, mild and milder peak days were also correctly forecasted in the correct range on days 126, 91, and 133 with 95% credible intervals of [125.07–125.53], [126.57–127] and [149.78–150.1] respectively. After the specified days, the peak time was accurately forecasted.

**Figure 5:**
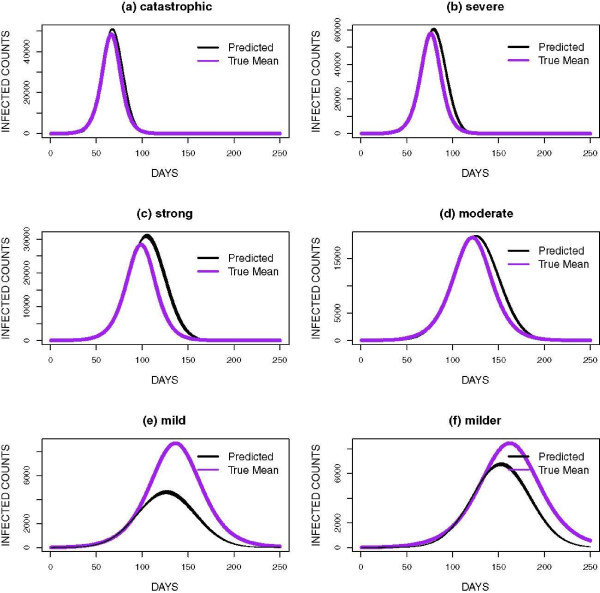
**Forecasts of peak times for each epidemic group.** The true mean curve and predicted curves are presented for: **(a)** catastrophic, **(b)** severe, **(c)** strong, **(d)** moderate, **(e)** mild and **(f)** milder. The results are presented based on epidemic peak time with the earliest peak presented first. These fits were made on days 77, 70, 91, 126, 91 and 133 for **(a-f)** respectively.

The mean curve for each of the groups and the predicted curves based on the last 400 MCMC iterations is shown in Figure 5 and the results are presented by peak time starting with the earliest peak. The epidemics were also scaled by the peak height since the aim was to forecast the timing of the peak not the peak height.

#### Identification of new epidemics

**Figure 6:**
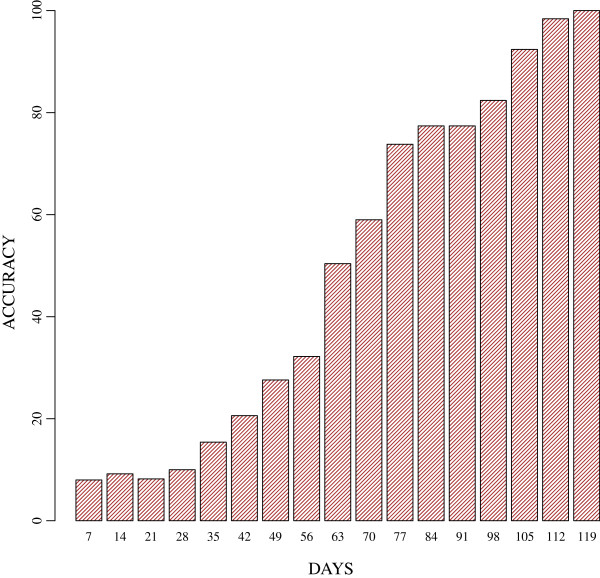
**Accuracy of identifying new epidemic curves.** The accuracy of identifying new epidemic curves. Accuracy is defined as the percentage of new epidemic curves, which are correctly identified as such.

The average accuracy based on identifying 600 epidemic curves different from those in the library is shown in Figure 6. Per Figure 6, the accuracy of identifying new epidemic curves increased rapidly as the epidemics neared their peaks. In addition, accuracy also consistently improved over time, reaching 100% on day 119. The classification of the known curves using the same value of *α* was similar to that observed in Figure 4.

### Forecast of peak time of epidemics in the U.S

Random forest correctly forecasted the peak time for 3/5, 2/5 and 3/5 of the seasonal epidemic curves given sixteen, twenty-five and thirty-three weeks of data, respectively. Similarly, the DP model correctly forecasted the peak time for 2/5, 3/5 and 3/5 of the epidemics in the test set given sixteen, twenty-five and thirty-three weeks of data, respectively. This suggests that the methods performed about the same in the forecasting of seasonal epidemics.

In contrast, the 2009–2010 epidemic curve that was observed during a pandemic peaked in week 42 of 2009, which was much earlier compared to the seasonal epidemics. Instead of evaluating accuracy based on prediction of the peak time, we focused on the accuracy of classification. The epidemic curve was incorrectly classified by random forest. Since random forest is a fully supervised classification algorithm, epidemic curves that are significantly different from those in the library will always be misclassified. The DP model identified the epidemic curve as being distinct from those in the library and was also able to accurately model the trend of the curve.

## Discussion

In this study we presented a Dirichlet process model for classifying epidemic curves and forecasting the expected peak time. The focus of this initial study was to establish the accuracy and usefulness of the method. We achieved our main objectives which were to: (i) compare the accuracy of the Dirichlet process model to that of random forest, (ii) forecast the epidemic peak time before the peak was observed and (iii) identify epidemic curves different from those in the library. We also observed that epidemics with higher transmission rates, implying higher *R* values, were easier to classify.

We acknowledge that there are limitations to using the proposed approach. For instance, using a parametric model to describe the epidemic curve might not be the most suitable option since the shape of the epidemic curve can vary from one epidemic to another. However, modeling the dynamics of epidemics using a nonparametric functional is a viable option (e.g. Gaussian processes, wavelets, or splines). In addition to limitations in the parametric model, inconsistencies in the accuracy of the DP model could also be attributed to the shape of the epidemic curves and the stochasticity inherent in MCMC methods. Since the DP model tries to capture the shape of the curve, it is also significantly influenced by the shape of the curve. In theory, one would expect the accuracy of the DP model to consistently improve with additional data. However, this is not always the case especially in the early stages of an epidemic, when the shape of the epidemic curve is not yet evident. Random forest on the other hand fails to take into account the shape of the curve and is therefore not heavily influenced by the variability introduced due to the shape of the curves.

The performance of random forest is likely due to its classification scheme. In this study, random forest classifies each epidemic curve one-thousand times and the final prediction is based on a voting process [29]. The epidemic group to which the new epidemic is classified the majority of the times is assigned the epidemic curve. Random forest also requires a shorter computation time compared to the DP model. This is because rather than considering every data point in the library and test set, each of the one-thousand classifications is based on a random sample of size  from each curve where *j* is the day of prediction. The number of classifications enables the method to capture different aspects of the curve by drawing different samples each time. Although the results seem to indicate that random forest predicts epidemic curves slightly better than the DP model, the performances are comparable especially in the classification of the real epidemics and epidemics simulated with high transmissibility (*R*) values.

## Conclusion

The results present situations in which the DP model performs well and situations in which it is likely to encounter problems. In most scenarios, the DP model encounters problems when distinguishing epidemic curves that are extremely similar or if the curve shape is not yet visible. However, there are several advantages to using the DP model over random forest. First, the DP model captures the shape and temporal structure which is not the case with random forest. This implies that under the random forest method, the data on day twenty can be moved to day five and the performance of random forest will not be affected. Second, classification algorithms such as random forest can suffer from the curse of dimensionality; the accuracy of the method depreciates as the dimensionality of the data increases. However this does not present an issue in this study. Third, the DP model does not only capture the trend of the epidemic but also the mean and variance of the curve. This information can be used to predict the peak time and the spread of the epidemic curves. Lastly, the DP model can also identify epidemics different from those in the library. However, since there are benefits to using both methods in prediction, an ensemble approach might be beneficial. In addition, classification using ILI data suggest that the performance of both methods could be improved. In future studies, we will explore ways to make the DP method more applicable to real-time forecasting of the epidemic curve.

## Competing interests

The authors declare that they have no competing interests.

## Authors’ contributions

EON and SCL carried out the analysis, and drafted the analysis. MVM provided the individual-based model, and participated in the design of the study. All authors read and approved the final manuscript.

## Pre-publication history

The pre-publication history for this paper can be accessed here:

http://www.biomedcentral.com/1471-2334/14/12/prepub

## Supplementary Material

Additional file 1 **Computational model and random forest description.** The file contains additional information on the construction of the computational epidemiology model and a brief description of random forest. Click here for file
