# Supplementary material for: A Dirichlet process model for classifying and forecasting epidemic curves
Source: BMC Infect Dis. 2014 Jan 9;14:12. doi: 10.1186/1471-2334-14-12 (PMC3901791; doi:10.1186/1471-2334-14-12)
Supplement: Additional file 1 — Computational model and random forest description. The file contains additional information on the construction of the computational epidemiology model and a brief description of random forest. [file 1471-2334-14-12-S1.pdf]

A Dirichlet Process Model for Classifying and Forecasting Epidemic Curves

Elaine O. Nsoesie*[1] , Scotland C. Leman [2] , and Madhav V. Marathe[1,3]

[1] Network Dynamics and Simulation Science Laboratory/Virginia Bioinformatics Institute/Virginia Tech, Blacksburg, Virginia, USA
[2] Department of Statistics/Virginia Tech, Blacksburg, Virginia, USA
[3] Department of Computer Science/ Virginia Tech, Blacksburg, Virginia, USA

Email: Elaine O. Nsoesie*- onelaine@vbi.vt.edu; Scotland C. Leman - leman@vt.edu; Madhav V. Marathe - mmarathe@vbi.vt.edu;

*Corresponding author

Computational Epidemiology Model

EpiFast is the individual-based approach used in this study and it was first described in Bisset et al. [1]. The approach is composed of two parts: (i) a time varying social contact network for modeling detailed contacts between individuals and (ii) a dynamical model that simulates the spatial spread of disease and effectiveness of public health interventions. The synthetic social contact network is constructed using various open source and commercially available data combined with social and behavioral theories. The synthetic social contact network of an urban population is a particular kind of random network that is statistically comparable to a realistic social contact network and preserves anonymity of individuals. To construct the network, first a synthetic population is created using an iterative proportional fitting technique. The synthetic population consists of synthetic people, with assigned demographical attributes based on data from the US census. Each individual is placed in a household and each household is located in a realistic geographical location such that when aggregated at the block group level, the synthetic population is statistically identical to the original census data [2–5] .

Next, each household is allotted activity templates by time of day based on several thousand responses to an activity or time-use survey for a specific region. The activity templates provide detailed description of activities for each household member throughout the day. Using a decision

tree based on demographics such as the number of workers in the household, number of children of various ages, etc., each synthetic household is matched and assigned the activity template of a household in the survey. Each activity performed by individuals in each household is assigned a location based on land-use patterns, tax data, etc., and the assigned locations are calibrated against data on travel-time distributions. These steps result in a synthetic population representing individuals and their activity patterns in a specified urban region. Synthetic individuals in the population interact with each other at various activity locations to produce realistic contact graphs where vertices represent individuals and edges represent contacts between individuals [6].

In addition to the time varying social contact network, a dynamical model that simulates spatial propagation of disease is also developed. The model is based on a Susceptible, Exposed, Infectious, Recovered (SEIR) representation. Individuals progress through the different disease states based on probabilistically timed incubating and infectiousness periods. The transition between disease states can be impacted by the attributes of the individuals (such as age, and health status) and the type of contact (casual, or intimate). The probability of transmission between susceptible (i) and infectious (j) individuals is given by:

$$p(w(i,j)) = 1 - (1-r)^{w(i,j)} \tag{1}$$

Here $w(i,j)$ represents the contact duration and r is the disease transmission rate, which is defined per sec/contact time. Each individual in the model has a separate disease model such that at each time step of a simulation, an individual is either susceptible, exposed, infectious, or recovered. Contacts between infectious and susceptible individuals at different activity locations result in disease transmission. Interventions such as vaccination, school closure, and other measures of social distancing are also implemented. Epidemics are simulated by selecting a synthetic contact network for a region, and setting initial conditions regarding the disease parameters and the number of initially infected individuals. Several studies have validated different components of the model. See [5], [2] and [13] for examples.

The approaches used in constructing this model can be found in several publications. See [7], and [8], and [9] for information on urban population mobility models. See [10], [11], [12], [13], and [14], for information on disease transmission models and the natural history of the disease. For further information on contact networks, see [12], [5], and [15].

Random Forest

Hastie et al. [16] define the random forest algorithm as follows:

1. For b = 1 to B:

(a) Draw a bootstrap sample Z* of size N from the training data

(b) Grow a random-forest tree T_b to the bootstrapped data, by recursively repeating the following steps for each terminal node of the tree, until the minimum node size n_{min} is reached

i. Select m variables at random from the p variables

ii. Pick the best variable/split-point among the m

iii. Split the node into two daughter nodes

2. Output the ensemble of trees ${(T_b)_1}^B$

To make a prediction at a new point x: Let $C_b(x)$ be the class prediction of the b_{th} random-forest tree. Then ${C^B}_{rf}(x)$ = majority vote ${(C_b(x))^B}_1$

Random Forest is an extension of bagging, an approach for combining several predictors to reduce the variance of an estimated prediction function [16, 17]. Advantages of random forest include efficiency on large databases and estimation of importance variables [18]. For the analysis in this paper, we used the randomForest package in R [19].

References

1. Bisset K, Chen J, Feng X, Kumar VSA, Marathe M: **EpiFast: a fast algorithm for large scale realistic epidemic simulations on distributed memory systems**. In *Proceedings of the 23rd international conference on Supercomputing*, ICS '09 2009:430–439.

2. Beckman R, Baggerly K, Mckay M: **Creating Synthetic Baseline Populations**. *Transportation Research Part A: Policy and Practice* 1996, **30**(6):415–429.

3. Speckman P, Vaughn K, Pas E: **Generating Household Activity-Travel Patterns (HATPs) for Synthetic Populations**. *Transportation Research Board 1997 Annual Meeting* 1997a.

4. Speckman P, Vaughn K, Pas E: **A Continuous Spatial Interaction Model: Application to Home-Work Travel in Portland, Oregon**. *Transportation Research Board 1997 Annual Meeting* 1997b.

5. Eubank S, Guclu H, Kumar VSA, Marathe M, Srinivasan A, Toroczkai Z, Wang N: **Modelling disease outbreaks in realistic urban social networks**. *Nature* 2004, **429**(6988):180–184.

6. Barrett C, Bisset K, Leidig J, Marathe A, Marathe M: **Economic and social impact of influenza mitigation strategies by demographic class**. *Epidemics* 2011, **3**:19–31.

7. Barrett C, Beckman R, Khan M, Kumar VSA, Marathe M, Stretz P, Dutta T, Lewis B: **Generation and analysis of large synthetic social contact networks**. In *Winter Simulation Conference*, WSC '09 2009:1003–1014.

8. TRBC: *5th-9th Biennial National Academies Transportation Research Board Conferences on Application Of Transportation Planning Methods* 1995-2003.

9. Bowman J, Bradley M, Shiftan Y, Lawton TK, Ben-Akiva M: **Demonstration of an activity based model system for Portland**. In *Proceedings of the 8th World Conference on Transport Research* 1998.

10. Bailey N: *The Mathematical Theory of Infectious Diseases and its Applications*. London: Griffin 1975.

11. Elveback L, Fox J, Ackerman E, Langworthy A, Boyd M, Gatewood L: *American Journal of Epidemiology* 1976, **103**(2):152–165.

12. Longini I, Nizam A, Xu S, Ungchusak K, Hanshaworakul W, Cummings D, Halloran E: **Containing pandemic influenza at the source**. *Science* 2005, **309**(5737):1083–1087.

13. Halloran ME, Ferguson N, Eubank S, Longini I, Cummings D, Lewis B, Xu S, Fraser C, Vullikanti A, Germann T, Wagener D, Beckman R, Kadau K, Barrett C, Macken C, Burke D, Cooley P: **Modeling targeted layered containment of an influenza pandemic in the United States**. *Proceedings of the National Academy of Sciences* 2008.

14. Hethcote HW: **The mathematics of infectious diseases**. *SIAM Review* 2000, **42**:599–653.

15. Newman M: **The structure and function of complex networks**. *SIAM Review* 2003, **45**:167–256.

16. Hastie T, Tibshirani R, Friedman J: *The Elements of Statistical Learning*. New York: Springer, corrected edition 2009.

17. Breiman L: **Bagging predictors**. *Machine Learning* 1996, **24**:123–140.

18. Breiman L: **Random Forests**. *Machine Learning* 2001, **45**:5–32.

19. Ihaka R, Gentleman R: **R: A Language for Data Analysis and Graphics**. *Journal of Computational and Graphical Statistics* 1996, (3):299–314.
